# Validation of NASH-CHECK: a novel patient-reported outcome measure for nonalcoholic steatohepatitis

**DOI:** 10.1186/s41687-023-00589-5

**Published:** 2023-07-14

**Authors:** James Twiss, Diane Whalley, Lynda Doward, Maria-Magdalena Balp, Clifford A. Brass, Donna Cryer, Arun Sanyal, Quentin M. Anstee

**Affiliations:** 1 RTI Health Solutions, Manchester, M20 2LS UK; 2 Novartis Pharma AG, Basel, Switzerland; 3 Novartis Pharmaceuticals Corporation, East Hanover, NJ USA; 4 Global Liver Institute, Washington, DC USA; 5 Virginia Commonwealth University, Richmond, VA USA; 6 Translational and Clinical Research Institute, Faculty of Medical Sciences, Newcastle University, Newcastle Upon Tyne, UK; 7 Newcastle NIHR Biomedical Research Centre, Newcastle Upon Tyne Hospitals NHS Trust, Newcastle Upon Tyne, UK

**Keywords:** Symptoms, Health-related quality of life (HRQOL), Reliability, Validity, Responsiveness

## Abstract

**Background:**

Standardized measures for evaluating patients’ experiences with nonalcoholic steatohepatitis (NASH) and their perceived changes with treatment in clinical trials have been limited. To meet this need, a patient-reported outcome (PRO) measure, NASH-CHECK, was developed to evaluate symptoms and health-related quality of life for patients with NASH. The objective of this study was to conduct a quantitative evaluation of the psychometric properties of NASH-CHECK.

**Methods:**

The study used data from a phase 2, randomized controlled trial of adult patients with NASH (NCT02855164). Analyses were conducted to determine the optimal scoring of NASH-CHECK and to evaluate reliability, construct validity, and ability to detect change in NASH-CHECK scale scores.

**Results:**

Data were available for 253 patients with NASH (61% female; mean [standard deviation] age = 53 [12] years). Following initial item-level analyses, including correlations and exploratory factor analysis, three items were removed from the measure. Confirmatory factor analysis supported the formation of four multi-item scales (Cognitive Symptoms, Activity Limitations, Social Impact, and Emotional Impact) and five single-item scales (Abdominal Pain, Abdominal Bloating, Fatigue, Sleep, and Itchy Skin). Psychometric analyses of the final NASH-CHECK scales provided support for their internal reliability, test–retest reliability, construct validity, and ability to detect change.

**Conclusion:**

The results support NASH-CHECK as a reliable, valid, and responsive measure to assess patients’ perspectives of symptoms and the health-related quality of life impact of NASH in clinical trials and in routine practice.

**Supplementary Information:**

The online version contains supplementary material available at 10.1186/s41687-023-00589-5.

## Background

Nonalcoholic fatty liver disease (NAFLD) is a global health challenge with detrimental impacts on mortality, morbidity, and health care resource utilization [1]. The progressive and most severe form of NAFLD, nonalcoholic steatohepatitis (NASH), was previously considered an asymptomatic disease in its early stages. However, recent evidence has shown the burdensome symptoms associated with NASH, including abdominal pain, fatigue, and cognitive impairments, can impair health-related quality of life (HRQOL) [2–4]. Clinical trials for NASH typically rely on histologic endpoints, hepatic imaging, or serologic biomarkers, which are not designed to collect data on the patient-perceived impact of NASH. Suitable patient-reported outcome (PRO) measures for NASH would enable evaluation of the symptoms and impacts of the condition from the patient’s perspective. Such information would add richness to data collected by key clinical endpoints in a trial setting or clinical practice and would provide valuable information to key stakeholders (e.g., clinicians, patients, regulators, reimbursement authorities, and policy makers) to support access to new pharmaceutical products and treatment pathways [5]. Previous research has shown that scores from PRO measures are weakly associated with clinical assessments in NASH, supporting the view that PROs capture unique information about the patient’s perspective [4, 6, 7].

Despite the potential for PROs to capture the broader impact of NASH, standardized, disease-specific measures for evaluating patients’ experiences with NASH have been either limited or not developed in accordance with key regulatory guidance for PRO measures [8–12]. NASH-CHECK, a novel, disease-specific PRO measure, was recently developed to evaluate symptoms and HRQOL for patients with NASH [13]. The development of this measure was guided by an international NASH-PRO Task Force composed of patient-centered outcomes researchers, clinical experts, patient advocacy advisors, and industry representatives. NASH-CHECK has been adopted as the PRO biomarker to inform the patient experience for the Liver Investigation: Testing Marker Utility in Steatohepatitis (LITMUS) program’s European NAFLD Registry [14]. The development of NASH-CHECK was consistent with best practice and regulatory guidance on the development and validation of PRO measures [8–11].

The initial development of NASH-CHECK included qualitative research conducted with patients with noncirrhotic NASH (fibrosis [F] levels F1 through F3) in the United States [13]. During this qualitative research phase, the content of NASH-CHECK was developed based on findings from concept elicitation interviews conducted with 23 patients with NASH. The patient-derived content focused on key symptoms (e.g., pain in the upper-right abdomen, fatigue, poor sleep quality, impaired memory, reduced focus) and HRQOL impacts (e.g., impaired physical functioning, reduced ability to conduct daily living tasks, self-consciousness, anxiety, low mood, reduced quality of relationships) of NASH. Cognitive debriefing interviews were subsequently conducted with 20 patients with NASH to confirm the content validity of NASH-CHECK. The qualitative development process resulted in a 31-item version of NASH-CHECK suitable for patients with noncirrhotic NASH (F1-F3).

The objective of this study was to conduct a psychometric evaluation of NASH-CHECK to confirm the optimal scale structure for the measure and to evaluate the psychometric properties (reliability, construct validity, and responsiveness) of the identified scales.

## Methods

### Study design and population

NASH-CHECK was included in an international phase 2, randomized, placebo-controlled, double-blind study (FLIGHT-FXR; NCT02855164) designed to assess the safety, tolerability, and efficacy of tropifexor in adult patients with noncirrhotic NASH [15, 16]. The study included patients from 17 countries across North and South America, Europe, Asia, and Australia. Language versions of NASH-CHECK for use in the trial were developed using industry standards for the translation and cultural adaptation of PRO measures [17]. The study population consisted of male and female patients aged 18 years or older who had NASH and liver fat of ≥ 10%. The psychometric evaluation of NASH-CHECK was conducted using data from two parts of the study: Part B was a 12-week study with 121 patients, and Part C was a 48-week study with 152 patients. For Part B, NASH was determined either by positive liver biopsy results obtained within 2 years before randomization confirming fibrosis level F1, F2, or F3, or by phenotypic diagnosis of NASH based on the presence of elevated alanine aminotransferase (ALT), type 2 diabetes mellitus, and high body mass index (BMI) [15]. For Part C, NASH was determined by positive biopsy results during screening or within 6 months before randomization that was consistent with NASH fibrosis F2 or F3 [16]. Psychometric analyses were conducted using data at baseline and week 12 from the Part B and Part C studies, as well as at weeks 2 and 48 from the Part C study.

### Study measures

The 31-item version of NASH-CHECK implemented in the FLIGHT-FXR study comprised 10 items assessing symptoms and 21 items assessing HRQOL. The measure has a recall period of 7 days. The symptoms items use 11-point numerical rating scales (NRSs) ranging from 0 (indicating no symptoms) to 10 (indicating worst possible or extreme symptoms). HRQOL items are grouped into activity limitations (8 items) and emotions and lifestyle (13 items), representing the preliminary conceptual groupings reflected in the conceptual model derived during the qualitative development process for NASH-CHECK [13]. The activity limitation items use a 5-point verbal rating scale ranging from “no difficulty” (scored 0) to “unable to do” (scored 4). The emotions and lifestyle items use a 4-point verbal rating scale ranging from “not at all” (scored 0) to “very much” (scored 3).

Additional trial measures used as supporting measures in the psychometric evaluation included a visual analog scale (VAS) for itch severity (24-h recall period; responses ranging from 0 = “no itch at all” to 10 = “the worst imaginable itch”), a VAS for sleep disturbance due to itch (24-h recall period; responses ranging from 0 = “no sleep loss” to 10 = “cannot sleep at all”), and a 6-point Patient Global Impression of Severity of NASH symptoms (PGIS; 7-day recall period; responses ranging from “no symptoms” to “very severe”). The Chronic Liver Disease Questionnaire (CLDQ) [18] and the VAS of the EQ-5D 5 Level version (EQ VAS) [19] were also used as supporting measures. The CLDQ is a 29-item, liver-specific measure with a 2-week recall, assessing HRQOL in six domains (abdominal symptoms, fatigue, systemic symptoms, activity, emotional function, and worry). CLDQ domain and total scores range from 1 (most impairment) to 7 (least impairment). The EQ VAS assesses current health status, with scores ranging from 0 (the worst health you can imagine) to 100 (the best health you can imagine). Clinical assessments used in the analyses included the presence of type 2 diabetes, BMI, fibrosis grade (Part C study only), NAFLD Activity Score (NAS), NAFLD Fibrosis score [20], enhanced liver fibrosis (ELF) score [21], AST level [22], ALT level [22], gamma-glutamyl transferase level [22], and hepatic fat percentage.

### Analysis methods

The psychometric evaluation of NASH-CHECK was conducted in two stages: first, item-level evaluations to inform item reduction and determine the optimal scale structure; and second, scale-level evaluations to assess the properties of the identified scales. For item-level analyses, the Part B and C data were analyzed separately. Most scale-level evaluations were conducted using the pooled Part B and C data, except for test–retest reliability (for which appropriate test–retest assessments were available only in Part C) and responsiveness (for which change from baseline to week 48 using Part C data was evaluated in addition to change from baseline to week 12 using the pooled Part B and C data). A summary of the data used for each analysis is presented in Additional file 1: Table S1.

Missing visit-level data were not imputed for any of the study measures. NASH-CHECK data were analyzed as observed, with no imputation of missing items. All other PRO measures were scored according to the respective instrument’s scoring guidelines.

#### Item analysis

Analysis of NASH-CHECK item scores was conducted using baseline data to determine the final items for inclusion in NASH-CHECK and the optimal scale structure for scoring. Assessments included floor effects (percentage of patients reporting no symptoms or HRQOL impacts [i.e., scoring 0]) and ceiling effects (percentage of patients reporting the most severe symptoms or HRQOL impacts [i.e., the maximum possible score]), in addition to item correlations. Inter-item correlations were computed using Pearson correlation for the symptom items and polychoric correlation for the remaining items. Exploratory factor analysis (EFA) was conducted using Part B baseline data to determine a preliminary scale structure. For patients with missing baseline data, their NASH-CHECK responses at either week 6 or week 12 were used to maximize sample size for EFA. Models retaining varying numbers of factors (based on the initial analysis and guided by the initial conceptual model) were evaluated. Eigenvalues > 1 were used to identify separate factors, and chi-square tests for differences between alternative factor solutions were used to guide selection of the best-fitting factor models.

Items were identified as candidates for removal from NASH-CHECK if they demonstrated redundancy (e.g., inter-item correlations > 0.8) or were poorly related to other items (e.g., inter-item correlations < 0.2 or EFA factor loadings < 0.3). A preliminary scale structure was determined based on the item-level analyses and subsequently confirmed using confirmatory factor analysis (CFA) using the Part C baseline data. Criteria to evaluate acceptable model fit were: chi-square test statistic P > 0.05; comparative fit index (CFI) ≥ 0.95; Tucker-Lewis index (TLI) ≥ 0.95; root mean square error of approximation (RMSEA) ≤ 0.06; standardized root mean square residual (SRMR) ≤ 0.08 (for symptom items); and weighted root mean square residual (WRMR) ≤ 1.0 (for HRQOL items) [23, 24].

#### Scale evaluation

The NASH-CHECK scales confirmed through CFA were evaluated to assess scale-level properties, including reliability, construct validity, and responsiveness.

For multi-item scales, internal consistency reliability was assessed by Cronbach’s coefficient alpha, with an optimal range considered to be 0.70 to 0.90 [25]. Estimates of McDonald’s omega coefficient [26, 27] were calculated using the CFA standardized estimates.

Test–retest reliability was assessed using NASH-CHECK scale scores at baseline and week 2 for two stable groups defined as (1) patients who had no change from baseline to week 2 on PGIS and (2) patients in the placebo treatment arm who had no change from baseline to week 2 on PGIS. Intraclass correlation coefficients (ICCs) were calculated using 2-way mixed-effects analysis of variance with absolute agreement for single measures [28]. An ICC of 0.70 or above was considered to indicate acceptable reliability [29].

Construct validity was evaluated via convergent and divergent correlations between NASH-CHECK scale scores and scores on the supporting measures. Pearson correlations were computed between NASH-CHECK scales and VAS for itch, VAS for sleep disturbance, CLDQ scales, EQ VAS, and clinical assessments; polyserial correlations were computed between NASH-CHECK scales and PGIS. The strength of the correlations was interpreted based on Cohen’s [30] criteria: correlations between 0.10 and 0.29 are considered small, correlations between 0.30 and 0.49 are considered moderate, and correlations of 0.50 or greater are considered strong. NASH-CHECK scale scores were hypothesized to correlate more strongly with measures assessing related concepts than with more disparate concepts. A full description of the a priori validation hypothesis is provided in the supplementary materials. Construct validity was also assessed by evaluating differences (using analysis of variance [ANOVA]) in mean scores between known groups based on PGIS.

Responsiveness was evaluated by comparing differences (using ANOVA) in the mean change from baseline in NASH-CHECK scale scores at week 12 and week 48 between patients categorized as improved, no change, or worsened on PGIS. Responsiveness was further evaluated through correlations between changes in NASH-CHECK scale scores and changes on supporting measures.

## Results

### Baseline characteristics

The analysis sample comprised a total of 253 patients with noncirrhotic NASH (104 patients from the Part B study and 149 patients from Part C). Baseline characteristics are shown in Table 1.

**Table 1:** Baseline patient characteristics

Baseline characteristics	Part B sample (n = 104)	Part C sample (n = 149)	Combined Part B and Part C sample (n = 253)
*Gender, n (%)*			
Male	47 (45.2)	52 (34.9)	99 (39.1)
Female	57 (54.8)	97 (65.1)	154 (60.9)
*Age, years*			
Mean (SD), min–max	51.2 (12.7), 19.0–79.0	54.9 (10.9), 25.0–79.0	53.4 (11.8), 19.0–79.0
*Race, n (%)*			
Caucasian	60 (58.3)	111 (74.5)	171 (67.9)
Not Caucasian	43 (41.7)	38 (25.5)	81 (32.1)
*Ethnicity, n (%)*			
Hispanic or Latino	22 (25.3)	48 (36.9)	70 (32.3)
Not Hispanic or Latino	65 (74.7)	82 (63.1)	147 (67.7)
*BMI*			
Mean (SD), min–max	32.5 (6.1), 20.9–49.3	34.9 (6.1), 24.0–53.4	33.9 (6.2), 20.9–53.4
*T2DM, n (%)*			
Yes	68 (65.4)	85 (58.6)	153 (61.4)
No	36 (34.6)	60 (41.4)	96 (38.6)
*Liver biopsy fibrosis grade, n (%)*			
F2	−	63 (42.3)	63 (42.3)
F3	−	86 (57.7)	86 (57.7)
*Additional clinical assessments, Mean (SD), min–max*			
NAFLD fibrosis score	− 1.3 (1.4), − 5.4–1.2	− 0.7 (1.2), − 4.2–1.7	− 0.9 (1.3), − 5.4–1.7
ELF score	9.4 (1.1), 3.3–11.7	9.9 (0.9), 8.2–13.1	9.7 (1.0), 3.3–13.1
NAS	4.6 (1.3), 1.0–7.0	6.0 (0.6), 4.0–7.0	5.6 (1.0), 1.0–7.0
ALT level, U/L	79.9 (41.1), 25.0–248.0	70.3 (38.7), 26.0–275.0	74.2 (39.9), 25.0–275.0
AST level, U/L	57.5 (32.6), 16.5–224.0	56.2 (28.2), 21.0–227.5	56.7 (30.0), 16.5–227.5
GGT level, U/L	73.3 (58.0), 12.0–385.0	67.4 (49.6), 15.5–400.5	69.8 (53.2), 12.0–400.5
Hepatic fat^ a^	20.0 (6.9), 10.2–36.7	18.9 (7.1), 10.2–42.8	19.3 (7.0), 10.2–42.8

*ALT* alanine aminotransferase, *AST* aspartate aminotransferase, *BMI* body mass index, *ELF* Enhanced liver fibrosis, *GGT* gamma-glutamyl transferase, *NAFLD* nonalcoholic fatty liver disease, *NAS* NAFLD Activity Score, *SD* standard deviation, *T2DM* type 2 diabetes mellitus, *U/L* units per liter

^a^Collected at screening

### Item analysis

#### Descriptive item scores

Missing data were minimal for NASH-CHECK in both Part B and Part C studies. Among patients who completed NASH-CHECK (i.e., who had at least 1 non-missing response on the measure), two participants had missing data: one patient in the Part B study (0.96%) had 25 missing individual items at week 12, and one patient in the Part C study (0.67%) had 21 missing individual items at week 48.

Descriptive statistics at baseline for the NASH-CHECK items are shown in Additional file 1: Tables S2 and S3 in the Supplementary Material. Scores for the NASH-CHECK items at baseline, as well as those for the other PRO measures, indicated that patients in the analysis sample experienced limited symptomatic impact. Although there were minimal ceiling effects for the NASH-CHECK items, floor effects (i.e., percentage of patients scoring 0, indicating best status) were observed across most NASH-CHECK items; for example, at baseline, floor effects ranged from 17.4% to 83.7% in the Part B study and from 14.8% to 77.9% in the Part C study. Other PRO measures in the trial showed similar floor effects; for example, 60–73% for VAS for itch; 71–85% for VAS for sleep disturbance; and 44–59% for PGIS.

#### Item correlations

Most NASH-CHECK items showed at least moderate correlations (≥ 0.3) with other items hypothesized to assess the same underlying measurement concept. Overall, the strongest correlations suggesting potential redundancy (i.e., ≥ 0.8) were generally among subsets of items assessing tiredness and fatigue, cognitive symptoms, daily activity limitations, ambulation, and relationship and social issues. In contrast, the item assessing food restriction had consistently lower correlations with other items.

#### Exploratory factor analysis

Factor loadings for the best-fitting EFA model solutions for symptoms, activity limitations, and emotions and lifestyle items are shown in Additional file 1: Table S4 in the Supplementary Material. Among the symptom items, the EFA results supported a 3-factor model (χ2[18, n = 103] = 23.89; P = 0.159; RMSEA = 0.06; CFI = 0.99, TLI = 0.97; SRMR = 0.03), including a 4-item Cognitive Symptoms scale that formed a clear and interpretable factor. Further interpretation of the results supported the remaining symptom items as single-item scales.

Among the HRQOL items, the EFA results supported the separation of the 8 activity limitations items and the 13 emotions and lifestyle items in a 2-factor model (χ2[169, n = 103] = 229.00; P = 0.002; RMSEA = 0.06; CFI = 0.99, TLI = 0.98; SRMR = 0.06). Separate EFA models were estimated to further explore each of these two groups of items.

For the activity limitations items, a 2-factor model comprising daily activities (4 items) and ambulation (4 items) subscales was the best-fitting solution (χ2[13, n = 103] = 16.37; P = 0.230; RMSEA = 0.05, CFI = 1.0, TLI = 1.0, SRMR = 0.02). However, the two factors were highly correlated (r = 0.85), indicating considerable overlap and potential support also for a single-factor solution.

For the emotions and lifestyle items, a 2-factor model comprising emotional impact (4 items) and social impact (7 items) subscales was the best-fitting solution (χ2[53, n = 103] = 57.82; P = 0.302; RMSEA = 0.03; CFI = 1.0, TLI = 1.0; SRMR = 0.06). However, high correlations between the 2 factors (r = 0.71) again indicated potential support also for the 1-factor solution. The analysis further indicated relatively low factor loadings for one item (food restriction), and another item (worry to family) had significant loadings onto multiple scales.

### Item reduction

Based on the item-level evaluations and further informed by qualitative data from the initial instrument development, three items were removed from NASH-CHECK: the item “need to rest” was considered redundant due to high inter-item correlations; the item “worry to family” was considered potentially multidimensional due to multiple loadings in EFA and relatively low inter-item correlations; and “food restriction” was considered unrelated to the intended concept due to relatively low inter-item correlations and EFA factor loadings. Removal of these items resulted in the final 28-item version of NASH-CHECK.

### Confirmatory factor analysis

The preliminary potential scales identified through EFA were evaluated through CFA using the Part C baseline data. Factor loadings for the best-fitting CFA model solutions for symptoms, activity limitations, and emotions and lifestyle items are shown in Table 2.

**Table 2:** Confirmatory factor analysis: standardized item factor loadings for NASH-CHECK scales

NASH-CHECK items (CFA model; n = 149)	Standardized factor loadings	
Cognitive symptoms items (1-factor model^ a^)	Cognitive symptoms	
Focusing	0.871*	
Thinking clearly	0.994*	
Following conversation	0.786*	
Forgetful	0.804*	

Activity limitations items (bifactor model)	Activity limitation (general factor)	Ambulation (specific group factor)
Bending	0.832*	–
Light chores	0.951*	–
Heavy chores	0.976*	–
Lifting	0.949*	–
Short walk	0.800*	0.451*
Long walk	0.741*	0.653*
Brisk walk	0.767*	0.511*
Walk up stairs	0.840*	0.240*

Emotions and social items (separate 1-factor models)	Emotional impact	Social impact
Worry	0.821*	
Feel down	0.956*	
Feel angry	0.761*	
Feeling judged	0.511*	
Relationships		0.849*
Everyday activities		0.955*
Family life		0.867*
Intimacy		0.708*
Socialise		0.777*
Spare time		0.993*
Work or study		0.834*

*CFA* confirmatory factor analysis, *CFI* comparative fit index, *NASH* nonalcoholic steatohepatitis, *RMSEA* root mean square error of approximation, *SRMR* standardized root mean square residual, *TLI* Tucker–Lewis index, *WRMR* weighted root mean square residual

^a^The 1-factor model allowed residuals between ‘Following conversation’ and ‘Forgetful’ items to correlate, as suggested by the modification indices and residual correlation matrix of the unadjusted model

^*^P < 0.05 for H0: Loading = 0

Overall model fit: Cognitive Symptoms: χ^2^[1, n = 149] = 0.000; *P* = 0.994; RMSEA = 0.00; CFI = 1.0, TLI = 1.0; SRMR = 0.00. Activity Limitations: χ2[16, n = 149] = 31.254; *P* = 0.013; RMSEA = 0.08; CFI = 1.0, TLI = 1.0; WRMR = 0.46. Explained common variance of the general factor = 0.86. Emotional Impact: χ2[1, n = 149] = 1.129; *P* = 0.288; RMSEA = 0.03; CFI = 1.0, TLI = 1.0; WRMR = 0.12. Social Impact: χ2[14, n = 149] = 27.526; *P* = 0.016; RMSEA = 0.08; CFI = 1.0, TLI = 1.0; WRMR = 0.61

For the four symptom items assessing cognitive symptoms (items 6–9: Focusing, Thinking Clearly, Following Conversation, Forgetful), CFA confirmed the single-factor solution with acceptable model fit (χ2[1, n = 149] = 0.000; P = 0.994; RMSEA = 0.00; CFI = 1.0, TLI = 1.0; SRMR = 0.00).

For the activity limitations items, the optimum CFA model was a bifactor model (χ2[16, n = 149] = 31.254; P = 0.013; RMSEA = 0.08; CFI = 1.0, TLI = 1.0; WRMR = 0.46); this model included a general factor containing all eight items, as well as a minor group factor comprising the subset of four items assessing ambulation. Although, the model indicated a degree of support for an ambulation item subset, the general factor was strong (explaining 86% of the variance), showing greater support for the overall activity limitation scale. Accordingly, all items were retained in a single activity limitations scale.

The optimum CFA models for the emotions and lifestyle items were two separate 1-factor models comprising the four emotional impact items (χ2[1, n = 149] = 1.129; P = 0.288; RMSEA = 0.03; CFI = 1.0, TLI = 1.0; WRMR = 0.12) and the seven social impact items (χ2[14, n = 149] = 27.526; P = 0.016; RMSEA = 0.08; CFI = 1.0, TLI = 1.0; WRMR = 0.61), respectively. The 1-factor model comprising all 11 emotions and lifestyle items from the EFA was not supported.

**Fig. 1:**
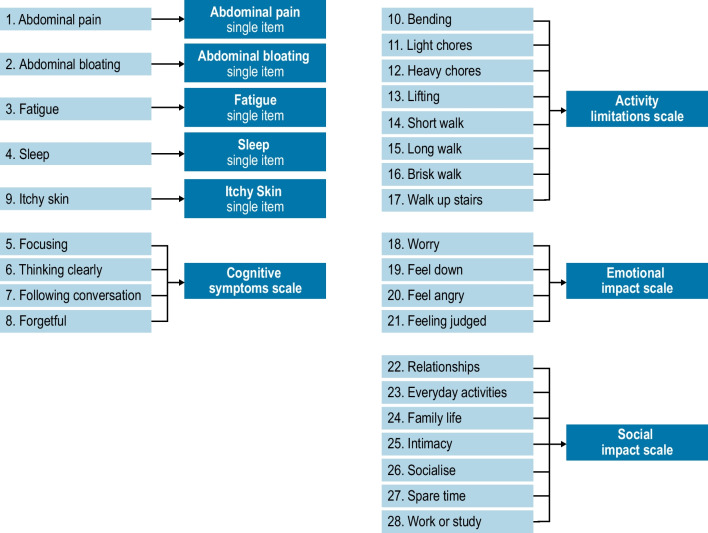
Final scale structure for NASH-CHECK symptoms and HRQOL items. *HRQOL* health-related quality of life

The final scale structure for NASH-CHECK is presented in Fig. 1. The final measure was scored as five single-item scales assessing Abdominal Pain, Abdominal Bloating, Fatigue, Sleep, and Itchy Skin, in addition to four multi-item scales: Cognitive Symptoms (4 items), Activity Limitations (8 items), Emotional Impact (4 items), and Social Impact (7 items). Scale scores were computed as the average score across the items comprising the scale and, for the three HRQOL scales, transformed to range from 0 to 10, with higher scores indicating more severe symptoms or greater HRQOL impact.

### Scale evaluation

#### Descriptive scale scores

Descriptive statistics for the NASH-CHECK scale scores at baseline are presented in Table 3. As found for the individual items, floor effects for the NASH-CHECK scale scores were consistent with those observed for other PRO assessments in the trial.

**Table 3:** Descriptive statistics for NASH-CHECK scale scores at baseline

NASH-CHECK scale score	Part B					Part C				
	n	Mean (SD)	Min–Max	Floor, n (%)	Ceiling, n (%)	n	Mean (SD)	Min–Max	Floor, n (%)	Ceiling, n (%)
Abdominal Pain	86	0.72 (1.47)	0.0–7.0	60 (69.8)	0 (0.0)	149	1.26 (1.96)	0.0–8.0	90 (60.4)	0 (0.0)
Abdominal Bloating	86	1.55 (2.12)	0.0–8.0	45 (52.3)	0 (0.0)	149	2.08 (2.62)	0.0–9.0	64 (43.0)	0 (0.0)
Fatigue	86	3.06 (2.55)	0.0–9.0	15 (17.4)	0 (0.0)	149	3.54 (2.99)	0.0–10.0	36 (24.2)	1 (0.7)
Sleep	86	1.77 (2.55)	0.0–9.0	40 (46.5)	0 (0.0)	149	2.66 (2.76)	0.0–10.0	49 (32.9)	1 (0.7)
Itchy Skin	86	1.28 (1.97)	0.0–10.0	46 (53.5)	1 (1.2)	149	1.57 (2.06)	0.0–9.0	67 (45.0)	0 (0.0)
Cognitive Symptoms	86	1.38 (1.82)	0.0–8.0	23 (26.7)	0 (0.0)	149	1.87 (2.16)	0.0–8.5	40 (26.8)	0 (0.0)
Activity Limitations	86	1.68 (2.05)	0.0–7.2	27 (31.4)	0 (0.0)	149	1.67 (1.93)	0.0–8.8	39 (26.2)	0 (0.0)
Emotional Impact	86	2.11 (1.66)	0.0–7.5	12 (14.0)	0 (0.0)	149	2.52 (1.96)	0.0–8.3	16 (10.7)	0 (0.0)
Social Impact	86	0.88 (1.40)	0.0–8.6	38 (44.2)	0 (0.0)	149	1.19 (1.72)	0.0–7.1	76 (51.0)	0 (0.0)

NASH-CHECK scores range from 0 to 10, with higher scores indicating worse symptoms/HRQOL

*HRQOL* health-related quality of life, *Max* maximum, *Min* minimum, *SD* standard deviation

#### Reliability

Cronbach’s coefficient alpha and estimates of McDonald’s omega for internal consistency of the NASH-CHECK multi-item scales were all above 0.70 (Table 4), indicating that the individual items are sufficiently related to form the intended scales. Cronbach’s coefficient alpha ranged from 0.77 (Emotional Impact) to 0.94 (Activity Limitations), and estimates of McDonald’s omega ranged from 0.79 (Emotional Impact) to 0.95 (Social Impact).

**Table 4:** Internal consistency and test–retest reliability coefficients for NASH-CHECK scales

NASH-CHECK scale score	Cronbach’s alpha^ a^	McDonald’s omega^b^	ICC (95% CI), n for PGIS stable group^ b^	ICC (95% CI), n for PGIS stable and placebo group^ b^
Abdominal pain	–	–	0.65 (0.50–0.76), 77	0.76 (0.54–0.88), 28
Abdominal bloating	–	–	0.80 (0.70–0.87), 77	0.88 (0.76–0.94), 28
Fatigue	–	–	0.70 (0.56–0.80), 77	0.70 (0.44–0.85), 28
Sleep	–	–	0.62 (0.46–0.74), 77	0.66 (0.39–0.83), 28
Itchy skin	–	–	0.40 (0.19–0.57), 77	0.65 (0.37–0.82), 28
Cognitive symptoms	0.92	0.88	0.77 (0.67–0.85), 77	0.84 (0.68–0.92), 28
Activity limitations	0.94	0.91	0.79 (0.68–0.86), 77	0.76 (0.55–0.88), 28
Emotional impact	0.77	0.79	0.68 (0.52–0.79), 77	0.79 (0.60–0.90), 28
Social impact	0.89	0.95	0.90 (0.85–0.94), 77	0.94 (0.88–0.97), 28

*CI* confidence interval, *ICC* intraclass correlation coefficient, *PGIS* Patient Global Impression of Severity

^a^Cronbach’s coefficient alpha was calculated for the NASH-CHECK multi-item scales at baseline

^b^McDonald’s omega coefficient was calculated for the NASH-CHECK multi-item scales at baseline using the CFA standardized estimates [33, 34]

^b^ICC was calculated from baseline to week 2 for a stable subsample defined as patients with no change in PGIS (PGIS change = 0) between baseline and week 2

For most NASH-CHECK scale scores, test–retest ICCs were above 0.70 (Table 4), indicating good reliability and showing that the scores remained stable over time when there had been no change in PGIS. ICCs were slightly lower for Abdominal Pain (0.65), Sleep (0.62), and Emotional Impact (0.68) scores and considerably lower for the Itchy Skin score (0.40). To evaluate whether the observed low ICC for the Itchy Skin score was due to a real change related to the known occurrence of pruritus among patients receiving active treatment in the trial, test–retest was re-evaluated using patients in the placebo group with no change in PGIS; the ICC for the Itchy Skin score based on this subsample was 0.65.

#### Construct validity

At baseline, moderate-to-strong correlations (|*r*|> 0.3) were observed between NASH-CHECK scale scores and scores on comparator measures assessing similar concepts, supporting the construct validity of the NASH-CHECK scales (Table 5). As predicted, strong correlations (> 0.5) were observed between the following at baseline:

- NASH-CHECK Abdominal Pain scores with CLDQ Abdominal Symptoms scores (|r|= 0.60)
- NASH-CHECK Abdominal Bloating scores with CLDQ Abdominal Symptoms scores (|r|= 0.74)
- NASH-CHECK Emotional Impact scores with CLDQ Worry scores (|r|= 0.74)
- NASH-CHECK Activity Limitations scores with CLDQ Activity scores (|r|= 0.66)
- NASH-CHECK Itchy skin with VAS for itch scores (|r|= 0.63)
- NASH-CHECK Sleep with VAS for sleep disturbance (|r|= 0.51).

**Table 5:** Construct validity correlations between NASH-CHECK and the supporting PRO measures at baseline

NASH-CHECK scale score	Correlation coefficient^ a^										
	Itch VAS	Sleep VAS	PGIS	CLDQ fatigue	CLDQ activity	CLDQ emotional function	CLDQ abdominal symptoms	CLDQ systemic symptoms	CLDQ worry	CLDQ total	EQ VAS
Abdominal Pain	0.25	0.28	0.55	− 0.41	− 0.37	− 0.37	− 0.60	− 0.44	− 0.26	− 0.49	− 0.16
Abdominal Bloating	0.19	0.26	0.62	− 0.58	− 0.55	− 0.50	− 0.74	− 0.59	− 0.28	− 0.65	− 0.27
Fatigue	0.15	0.23	0.65	− 0.75	− 0.48	− 0.59	− 0.55	− 0.59	− 0.39	− 0.67	− 0.38
Sleep	0.20	0.51	0.52	− 0.50	− 0.38	− 0.65	− 0.41	− 0.47	− 0.33	− 0.54	− 0.29
Itchy Skin	0.63	0.47	0.48	− 0.35	− 0.30	− 0.39	− 0.39	− 0.51	− 0.23	− 0.43	− 0.21
Cognitive Symptoms	0.20	0.29	0.61	− 0.71	− 0.54	− 0.66	− 0.58	− 0.61	− 0.31	− 0.68	− 0.39
Activity Limitations	0.07	0.29	0.48	− 0.64	− 0.66	− 0.60	− 0.55	− 0.68	− 0.39	− 0.70	− 0.49
Emotional Impact	0.10	0.17	0.34	− 0.48	− 0.44	− 0.56	− 0.43	− 0.46	− 0.74	− 0.62	− 0.36
Social Impact	0.14	0.28	0.43	− 0.58	− 0.49	− 0.59	− 0.42	− 0.55	− 0.52	− 0.63	− 0.44

*CLDQ* Chronic Liver Disease Questionnaire, *PGIS* Patient Global Impression of Severity, *PRO* patient-reported outcome, *VAS* visual analog scale

^a^Pearson correlation for itch VAS, sleep disturbance VAS, CLDQ scales, and EQ VAS; polyserial correlation for PGIS

In addition, moderate-to-strong correlations were observed between all NASH-CHECK scale scores and PGIS at baseline and between most NASH-CHECK scales and EQ VAS. As anticipated, correlations between NASH-CHECK scale scores and clinical assessments were small (|*r*|< 0.3), with most correlations less than 0.1 (Additional file 1: Table S5 in the Supplementary Material). Correlations between CLDQ scale scores and the clinical measures were similarly small (correlation coefficients, |r|, ranged from 0.00 [CLDQ Emotional Function and ELF score] to 0.12 [CLDQ Systemic Symptoms and NAS]). These findings confirmed a weak association between the PRO and clinical assessments in the study.

**Fig. 2:**
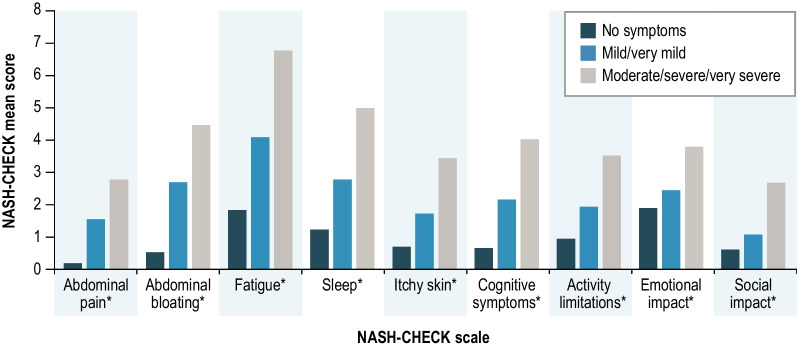
NASH-CHECK Baseline Scores by PGIS (n = 235). *P < 0.0001. *PGIS* Patient Global Impression of Severity

NASH-CHECK scale scores discriminated significantly between groups according to the PGIS at baseline (Fig. 2) (*P* < 0.0001 for all scales), confirming known-groups validity. All pairwise comparisons (27 pairwise tests conducted) between PGIS groups were also statistically significant (p < 0.05). As expected, patients who reported more severe NASH symptoms on the PGIS had higher mean NASH-CHECK scores than those reporting less severe symptoms. Furthermore, significant differences in mean NASH-CHECK scale scores between PGIS groups continued to be observed when BMI was included as a covariate (*P* < 0.0001 for all scales), confirming the ability of NASH-CHECK scores to discriminate between NASH symptom severity groups irrespective of BMI.

#### Responsiveness

**Fig. 3:**
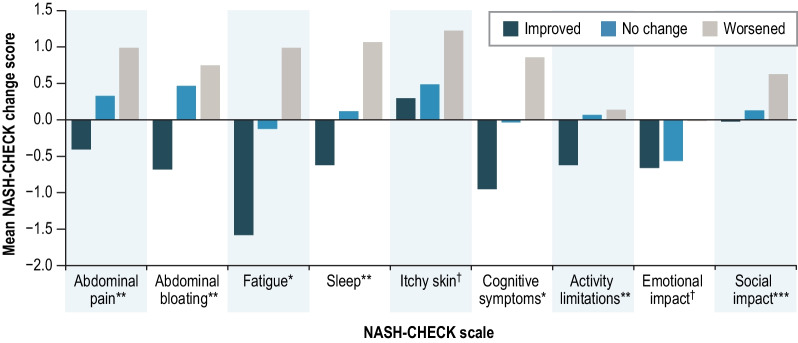
NASH-CHECK change from baseline scores by PGIS change at week 12 (n = 216). *P < 0.0001; **P < 0.01; ***P < 0.05; ^†^P > 0.05. *PGIS* Patient Global Impression of Severity. *Notes*: Improved = PGIS improvement ≥ 1 point; no change = PGIS 0 point change; worsened = PGIS worsening ≥ 1 point

**Fig. 4:**
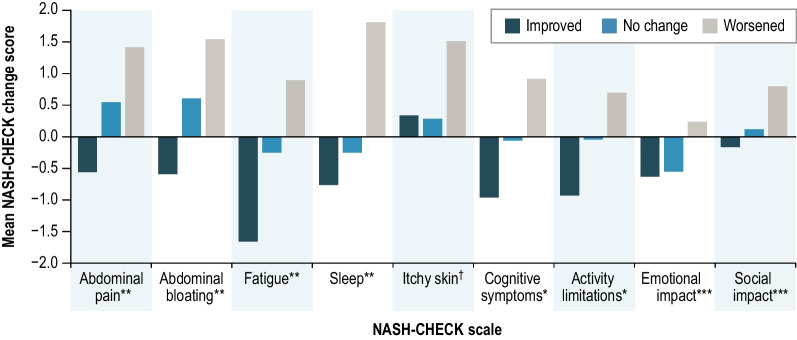
NASH-CHECK change from baseline scores by PGIS change at week 48 (n = 116). *P < 0.0001; **P < 0.01; ***P < 0.05; ^†^P > 0.05. *PGIS* Patient Global Impression of Severity. *Notes*: improved = PGIS improvement ≥ 1 point; no change = PGIS 0-point change; worsened = PGIS worsening ≥ 1 point

Mean change from baseline in NASH-CHECK scale scores at week 12 and week 48 by change based on PGIS (improved, no change, worsened) are presented in Figs. 3 and 4, respectively. Differences in mean change scores were observed for all NASH-CHECK scales (P < 0.05), except for Itchy Skin at weeks 12 and 48 and Emotional Impact at week 12. Most pairwise comparisons between PGIS groups were also statistically significant (P < 0.05 for 20 out of 27 pairwise tests from baseline to week 12, and 19 out of 27 pairwise tests from baseline to week 48). The pattern of NASH-CHECK change scores across groups was as expected for most scales, with the highest negative change scores (indicating improvement) among patients who had improved on PGIS and the highest positive change scores (indicating deterioration) among patients who had worsened. The pattern of correlations between change in NASH-CHECK scale scores and change in scores on supporting PRO and clinical measures (see Additional file 1: Table S6 in the Supplementary Material) was as expected and similar to the cross-sectional correlations.

## Discussion

NASH-CHECK is a novel, NASH-specific measure that was developed in accordance with best practice guidance on the development and validation of PRO measures [8–11]. Building on previous qualitative research to develop and establish content validity for the measure [13], this study sought to establish its quantitative measurement properties using data from a phase 2, multi-part, randomized controlled trial (FLIGHT-FXR) [15, 16]. Item-level analyses informed identification of the final 28 items of NASH-CHECK and confirmed the optimal grouping of items to provide scale scores. The identified scale structure corresponded to the conceptual groupings hypothesized based on the initial model derived during the initial qualitative development process [13]. Scale-level analyses supported the psychometric properties of the identified NASH-CHECK scale scores. Internal consistency reliability was adequate for the four multi-item scales, indicating that the items are sufficiently related to form scales. Following identification of a stable sample, test–retest reliability coefficients were above or approximate to the recommended level to indicate that NASH-CHECK scores are adequately stable over time. ICCs were slightly lower for three subscale scores (Abdominal Pain, Sleep, and Emotional Impact) and considerably lower for the Itchy Skin score when the stable sample was defined based on PGIS group alone. Due to the known increase in pruritis during the FLIGHT-FXR study, test–retest was also evaluated restricting the sample to patients in the placebo treatment arm with no change in PGIS. Although this resulted in higher ICC values for most of the NASH-CHECK scales, reliability of the Sleep and Itchy Skin scale scores remained slightly below the recommended level to indicate adequate stability. Further evaluation of test–retest reliability in alternative samples would be useful to investigate this further.

The patterns of correlations between NASH-CHECK scale scores and scores on comparator measures were as expected, providing support that the NASH-CHECK scales measure their intended concepts. As expected, all NASH-CHECK scores discriminated between patients according to perceived symptom severity, further supporting the construct validity of NASH-CHECK. Furthermore, this association persisted when controlling for BMI, indicating that the relationship between NASH-CHECK scores and NASH symptom severity was not a function of obesity status. NASH-CHECK scores were also able to detect change associated with change in PGIS score over periods of 12 and 48 weeks, and changes in NASH-CHECK scores were correlated as expected with other PRO change scores. These findings suggest that NASH-CHECK will be valuable for quantifying change in patients’ experiences with NASH.

Low levels of associations were found between NASH-CHECK scale scores and clinical assessments such as NAS, ELF score, and ALT level. The observed correlations were comparable to those between CLDQ scale scores and the same clinical variables in this study and are consistent with previous research that has demonstrated only weak associations between PRO scores and clinical outcomes in NASH [4, 6, 7]. The reason for the low associations between clinical outcomes in NASH and PRO scores in unclear. However, given the currently available clinical markers, it is challenging to categorize patients into different stages of NAFLD on the basis of clinical outcomes and to stage fibrosis severity. Liver biopsy remains the established though imperfect reference standard for definitive diagnosis of the spectrum of NAFLD disease; however, it is invasive, resource intensive, and prone to sampling error [31]. Other noninvasive markers of cellular injury are commonly evaluated in NASH, such as aspartate aminotransferase (AST), alanine aminotransferase (ALT), or gamma-glutamyl transpeptidase (GammaGT). Although these variables are often elevated in patients with NASH, they might be minimal or even absent in advanced disease [32].

The weak associations between NASH-CHECK and the clinical outcomes suggest that PRO data capture unique information about the patient experience that is not available from histologic endpoints or serologic biomarkers for NASH. Such information is valuable in clinical trial settings to determine the broader impacts of NASH and its treatment from the patient perspective; this information provides regulators, policy makers, health technology assessment (HTA) authorities, and clinicians with important insights about the patients’ experience. NASH-CHECK also provides clinicians with a way to quantify the impact of NASH from the patient perspective that could be useful in clinical practice.

A limitation of this study was the relatively small sample size available for EFA (n = 103). However, the preliminary scales identified through EFA were confirmed through CFA in a separate sample, thus increasing confidence in the robustness of the identified scale structure. Evaluations of the longitudinal properties of NASH-CHECK were limited by the relatively low levels of symptoms and HRQOL impairments in both study samples at baseline, thus reducing the potential for change. Despite this, changes in NASH-CHECK scale scores were associated with changes in other PRO measures and most differed as expected across levels of change in PGIS.

## Conclusions

NASH-CHECK is a novel PRO measure developed specifically for use with patients with NASH. The scale structure identified in this study is consistent with the conceptual model originally developed from the literature and qualitative research conducted with patients with NASH. Scale scores derived from the measure are reliable, valid, and able to detect change. The results of this psychometric evaluation suggest NASH-CHECK is a valuable tool to capture patients’ experiences with the symptoms of NASH and its impact on HRQOL in clinical trials and in routine practice. NASH-CHECK is free for use and can be accessed via the authorized distributor at https://lifesciences.rws.com/nash-check?hsLang=en.

## Supplementary Information

**Additional file 1.** Supplementary Tables.

## Data Availability

Data are not available.

## Abbreviations

ALT: Alanine aminotransferase
ANOVA: Analysis of variance
AST: Aspartate aminotransferase
BMI: Body mass index
CFA: Confirmatory factor analysis
CFI: Comparative fit index
CI: Confidence interval
CLDQ: Chronic Liver Disease Questionnaire
EFA: Exploratory factor analysis
ELF: Enhanced liver fibrosis
GGT: Gamma-glutamyl transferase
HRQOL: Health-related quality of life
ICC: Intraclass correlation coefficient
NAFLD: Nonalcoholic fatty liver disease
NAS: NAFLD Activity Score
NASH: Nonalcoholic steatohepatitis
NRS: Numeric rating scale
PGIS: Patient Global Impression of Severity
PRO: Patient-reported outcome
RMSEA: Root mean square error of approximation
SD: Standard deviation
SEM: Standard error of the mean;
SRMR: Standardized root mean square residual
T2DM: Type 2 diabetes mellitus;
TLI: Tucker–Lewis index
VAS: Visual analog scale
WRMR: Weighted root mean square residual
