# Supplementary material for: Validation of NASH-CHECK: a novel patient-reported outcome measure for nonalcoholic steatohepatitis
Source: J Patient Rep Outcomes. 2023 Jul 14;7:69. doi: 10.1186/s41687-023-00589-5 (PMC10349018; doi:10.1186/s41687-023-00589-5)
Supplement: Supplementary file 1 — Additional file 1. Supplementary Tables. [file 41687_2023_589_MOESM1_ESM.docx]

# Supplementary Material

**Convergent and Divergent Correlations A Priori Hypotheses**

The following relationships were hypothesized:

- Moderate-to-strong correlations between NASH-CHECK scales and those subscales of the CLDQ that measure related concepts
- Moderate-to-strong correlations between NASH-CHECK scales and the PGIS
- Moderate correlations between the NASH-CHECK scales and the EQ VAS
- Strong positive correlations between:
  - VAS for itch and the NASH-CHECK itch item
  - VAS for sleep disturbance and the NASH-CHECK sleep item
- Strong correlations between:
  - CLDQ abdominal symptoms scale and the NASH-CHECK abdominal symptoms items
  - CLDQ activity scale and the NASH-CHECK activity limitations items
- No correlation or weak correlations between clinical outcomes (e.g., ALT and/or AST) and the NASH-CHECK scales.

Table S1. Overview of Data Used in the Psychometric Evaluation of NASH-CHECK

|  |  | Phase 2 Study^a^ | | |
| --- | --- | --- | --- | --- |
| Analysis/Property | Purpose | Part B  (n = 104) | Part C  (n = 149) | Pooled Parts B and C  (n = 253) |
| Item Reduction and Scale Structure | |  |  |  |
| Item distributional properties | Evaluate item-level floor and ceiling effects | ✔ | ✔ |  |
| Inter-item correlations | Evaluate internal item associations | ✔ | ✔ |  |
| Exploratory factor analysis | Evaluate internal item associations; establish item groupings to inform preliminary scale structure | ✔ |  |  |
| Confirmatory factor analysis | Confirm scale structure |  | ✔ |  |
| Scale Evaluation | |  |  |  |
| Distributional properties | Evaluate scale-level floor and ceiling effects |  |  | ✔ |
| Internal consistency (Cronbach’s coefficient alpha and McDonald’s omega coefficient) | Evaluate the extent to which individual items in a multi-item scale are associated and capable of supporting a composite score |  |  | ✔ |
| Test-retest reliability (ICC) | Evaluate the reproducibility of scores over time among “stable” patients |  | ✔ |  |
| Convergent and divergent validity (correlations) | Evaluate associations among multiple indicators of similar and dissimilar constructs and the degree to which they follow predictable patterns |  |  | ✔ |
| Known-groups validity (ANOVA) | Compare scores across hypothesized subgroups of interest to provide support for discriminating ability |  |  | ✔ |
| Responsiveness (correlations and ANOVA) | Evaluate the extent to which scores can detect change in patients who have changed in the construct measured |  | ✔ | ✔ |

ANOVA = analysis of variance; ICC = intraclass correlation coefficient

Note: Analyses were conducted using NASH-CHECK data from baseline and week 12 for Part B; baseline, week 2, week 12, and week 48 for Part C; and baseline and week 12 for pooled Parts B and C.

^a^ [NCT02855164](https://clinicaltrials.gov/ct2/show/NCT02855164)

Table S2. NASH-CHECK Item Statistics at Baseline: Part B (N = 86)

| **NASH-CHECK item** | **Item Response, n (%)** | | | | | | | | | | | | | | | | | | | |
| --- | --- | --- | --- | --- | --- | --- | --- | --- | --- | --- | --- | --- | --- | --- | --- | --- | --- | --- | --- | --- |
| **Symptom items** | **0** | | **1** | **2** | | | **3** | **4** | | **5** | | **6** | | **7** | **8** | | | **9** | **10** | **Missing** |
| Item 1. Pain | 60 (69.8) | | 12 (14.0) | 6 (7.0) | | | 1 (1.2) | 3 (3.5) | | 2 (2.3) | | 1 (1.2) | | 1 (1.2) | 0 (0.0) | | | 0 (0.0) | 0 (0.0) | 18 (17.3) |
| Item 2. Bloating | 45 (52.3) | | 9 (10.5) | 10 (11.6) | | | 6 (7.0) | 4 (4.7) | | 6 (7.0) | | 3 (3.5) | | 2 (2.3) | 1 (1.2) | | | 0 (0.0) | 0 (0.0) | 18 (17.3) |
| Item 3. Fatigue | 15 (17.4) | | 13 (15.1) | 16 (18.6) | | | 12 (14.0) | 5 (5.8) | | 9 (10.5) | | 2 (2.3) | | 9 (10.5) | 3 (3.5) | | | 2 (2.3) | 0 (0.0) | 18 (17.3) |
| Item 4. Rest | 20 (23.3) | | 15 (17.4) | 15 (17.4) | | | 9 (10.5) | 6 (7.0) | | 3 (3.5) | | 4 (4.7) | | 7 (8.1) | 3 (3.5) | | | 2 (2.3) | 2 (2.3) | 18 (17.3) |
| Item 5. Sleep | 40 (46.5) | | 15 (17.4) | 11 (12.8) | | | 5 (5.8) | 3 (3.5) | | 3 (3.5) | | 0 (0.0) | | 1 (1.2) | 6 (7.0) | | | 2 (2.3) | 0 (0.0) | 18 (17.3) |
| Item 6. Focusing | 41 (47.7) | | 14 (16.3) | 10 (11.6) | | | 9 (10.5) | 4 (4.7) | | 1 (1.2) | | 2 (2.3) | | 3 (3.5) | 1 (1.2) | | | 1 (1.2) | 0 (0.0) | 18 (17.3) |
| Item 7. Thinking clearly | 47 (54.7) | | 18 (20.9) | 5 (5.8) | | | 3 (3.5) | 6 (7.0) | | 0 (0.0) | | 2 (2.3) | | 2 (2.3) | 3 (3.5) | | | 0 (0.0) | 0 (0.0) | 18 (17.3) |
| Item 8. Follow conversation | 58 (67.4) | | 15 (17.4) | 3 (3.5) | | | 4 (4.7) | 2 (2.3) | | 1 (1.2) | | 1 (1.2) | | 0 (0.0) | 2 (2.3) | | | 0 (0.0) | 0 (0.0) | 18 (17.3) |
| Item 9. Forgetful | 27 (31.4) | | 22 (25.6) | 14 (16.3) | | | 9 (10.5) | 3 (3.5) | | 3 (3.5) | | 2 (2.3) | | 3 (3.5) | 1 (1.2) | | | 2 (2.3) | 0 (0.0) | 18 (17.3) |
| Item 10. Itchy skin | 46 (53.5) | | 12 (14.0) | 12 (14.0) | | | 8 (9.3) | 2 (2.3) | | 0 (0.0) | | 3 (3.5) | | 2 (2.3) | 0 (0.0) | | | 0 (0.0) | 1 (1.2) | 18 (17.3) |
| **Activity items** | | **No difficulty** | | | **Mild difficulty** | | | | **Moderate difficulty** | | | | **Severe difficulty** | | | | **Unable to do** | | | **Missing** |
| Item 11. Bending | | 48 (55.8) | | | 23 (26.7) | | | | 10 (11.6) | | | | 4 (4.7) | | | | 1 (1.2) | | | 18 (17.3) |
| Item 12. Light chores | | 59 (68.6) | | | 21 (24.4) | | | | 2 (2.3) | | | | 3 (3.5) | | | | 1 (1.2) | | | 18 (17.3) |
| Item 13. Heavy chores | | 51 (59.3) | | | 23 (26.7) | | | | 6 (7.0) | | | | 6 (7.0) | | | | 0 (0.0) | | | 18 (17.3) |
| Item 14. Heavy objects | | 48 (55.8) | | | 19 (22.1) | | | | 11 (12.8) | | | | 5 (5.8) | | | | 3 (3.5) | | | 18 (17.3) |
| Item 15. Short walk | | 64 (74.4) | | | 11 (12.8) | | | | 10 (11.6) | | | | 0 (0.0) | | | | 1 (1.2) | | | 18 (17.3) |
| Item 16. Long walk | | 46 (53.5) | | | 23 (26.7) | | | | 10 (11.6) | | | | 3 (3.5) | | | | 4 (4.7) | | | 18 (17.3) |
| Item 17. Brisk walk | | 44 (51.2) | | | 21 (24.4) | | | | 12 (14.0) | | | | 5 (5.8) | | | | 4 (4.7) | | | 18 (17.3) |
| Item 18. Walk up | | 47 (54.7) | | | 21 (24.4) | | | | 10 (11.6) | | | | 7 (8.1) | | | | 1 (1.2) | | | 18 (17.3) |
| **Emotion and lifestyle items** | | **Not at all** | | | | **A little** | | | | | **Quite a lot** | | | | | **Very much** | | | | **Missing** |
| Item 19. Worry | | 15 (17.4) | | | | 47 (54.7) | | | | | 17 (19.8) | | | | | 7 (8.1) | | | | 18 (17.3) |
| Item 20. Feel down | | 42 (48.8) | | | | 38 (44.2) | | | | | 5 (5.8) | | | | | 1 (1.2) | | | | 18 (17.3) |
| Item 21. Feel angry | | 57 (66.3) | | | | 24 (27.9) | | | | | 3 (3.5) | | | | | 2 (2.3) | | | | 18 (17.3) |
| Item 22. Feel judged | | 62 (72.1) | | | | 20 (23.3) | | | | | 3 (3.5) | | | | | 1 (1.2) | | | | 18 (17.3) |
| Item 23. Relationships | | 72 (83.7) | | | | 12 (14.0) | | | | | 1 (1.2) | | | | | 1 (1.2) | | | | 18 (17.3) |
| Item 24. Everyday activities | | 66 (76.7) | | | | 18 (20.9) | | | | | 0 (0.0) | | | | | 2 (2.3) | | | | 18 (17.3) |
| Item 25. Family life | | 72 (83.7) | | | | 12 (14.0) | | | | | 2 (2.3) | | | | | 0 (0.0) | | | | 18 (17.3) |
| Item 26. Worry to family | | 46 (53.5) | | | | 35 (40.7) | | | | | 2 (2.3) | | | | | 3 (3.5) | | | | 18 (17.3) |
| Item 27. Intimacy | | 68 (79.1) | | | | 13 (15.1) | | | | | 3 (3.5) | | | | | 2 (2.3) | | | | 18 (17.3) |
| Item 28. Socialise | | 63 (73.3) | | | | 19 (22.1) | | | | | 4 (4.7) | | | | | 0 (0.0) | | | | 18 (17.3) |
| Item 29. Spare time | | 65 (75.6) | | | | 19 (22.1) | | | | | 1 (1.2) | | | | | 1 (1.2) | | | | 18 (17.3) |
| Item 30. Work or study | | 64 (74.4) | | | | 19 (22.1) | | | | | 2 (2.3) | | | | | 1 (1.2) | | | | 18 (17.3) |
| Item 31. Food restriction | | 28 (32.6) | | | | 40 (46.5) | | | | | 13 (15.1) | | | | | 5 (5.8) | | | | 18 (17.3) |

NASH = non-alcoholic steatohepatitis.

Table S3. NASH-CHECK Item Statistics at Baseline: Part C (N = 149)

| **NASH-CHECK Item** | **Item Response, n (%)** | | | | | | | | | | | | | | | | | | | |
| --- | --- | --- | --- | --- | --- | --- | --- | --- | --- | --- | --- | --- | --- | --- | --- | --- | --- | --- | --- | --- |
| **Symptom items** | **0** | | **1** | **2** | | | **3** | **4** | | **5** | | **6** | | **7** | **8** | | | **9** | **10** | **Missing** |
| Item 1. Pain | 90 (60.4) | | 15 (10.1) | 12 (8.1) | | | 9 (6.0) | 4 (2.7) | | 12 (8.1) | | 5 (3.4) | | 1 (0.7) | 1 (0.7) | | | 0 (0.0) | 0 (0.0) | 0 (0.0) |
| Item 2. Bloating | 64 (43.0) | | 24 (16.1) | 14 (9.4) | | | 9 (6.0) | 7 (4.7) | | 10 (6.7) | | 5 (3.4) | | 8 (5.4) | 5 (3.4) | | | 3 (2.0) | 0 (0.0) | 0 (0.0) |
| Item 3. Fatigue | 36 (24.2) | | 13 (8.7) | 14 (9.4) | | | 15 (10.1) | 21 (14.1) | | 7 (4.7) | | 9 (6.0) | | 12 (8.1) | 14 (9.4) | | | 7 (4.7) | 1 (0.7) | 0 (0.0) |
| Item 4. Rest | 39 (26.2) | | 23 (15.4) | 11 (7.4) | | | 14 (9.4) | 10 (6.7) | | 9 (6.0) | | 12 (8.1) | | 12 (8.1) | 11 (7.4) | | | 6 (4.0) | 2 (1.3) | 0 (0.0) |
| Item 5. Sleep | 49 (32.9) | | 20 (13.4) | 15 (10.1) | | | 18 (12.1) | 7 (4.7) | | 14 (9.4) | | 5 (3.4) | | 10 (6.7) | 6 (4.0) | | | 4 (2.7) | 1 (0.7) | 0 (0.0) |
| Item 6. Focusing | 67 (45.0) | | 15 (10.1) | 15 (10.1) | | | 12 (8.1) | 9 (6.0) | | 8 (5.4) | | 10 (6.7) | | 8 (5.4) | 5 (3.4) | | | 0 (0.0) | 0 (0.0) | 0 (0.0) |
| Item 7. Thinking clearly | 76 (51.0) | | 13 (8.7) | 13 (8.7) | | | 9 (6.0) | 12 (8.1) | | 8 (5.4) | | 10 (6.7) | | 5 (3.4) | 3 (2.0) | | | 0 (0.0) | 0 (0.0) | 0 (0.0) |
| Item 8. Follow conversation | 90 (60.4) | | 16 (10.7) | 10 (6.7) | | | 5 (3.4) | 11 (7.4) | | 10 (6.7) | | 3 (2.0) | | 2 (1.3) | 1 (0.7) | | | 0 (0.0) | 1 (0.7) | 0 (0.0) |
| Item 9. Forgetful | 53 (35.6) | | 25 (16.8) | 18 (12.1) | | | 13 (8.7) | 13 (8.7) | | 7 (4.7) | | 5 (3.4) | | 6 (4.0) | 6 (4.0) | | | 3 (2.0) | 0 (0.0) | 0 (0.0) |
| Item 10. Itchy skin | 67 (45.0) | | 26 (17.4) | 19 (12.8) | | | 12 (8.1) | 13 (8.7) | | 2 (1.3) | | 2 (1.3) | | 5 (3.4) | 2 (1.3) | | | 1 (0.7) | 0 (0.0) | 0 (0.0) |
| **Activity items** | | **No difficulty** | | | **Mild difficulty** | | | | **Moderate difficulty** | | | | **Severe difficulty** | | | | **Unable to do** | | | **Missing** |
| Item 11. Bending | | 84 (56.4) | | | 38 (25.5) | | | | 20 (13.4) | | | | 6 (4.0) | | | | 1 (0.7) | | | 0 (0.0) |
| Item 12. Light chores | | 102 (68.5) | | | 32 (21.5) | | | | 11 (7.4) | | | | 4 (2.7) | | | | 0 (0.0) | | | 0 (0.0) |
| Item 13. Heavy chores | | 82 (55.0) | | | 42 (28.2) | | | | 16 (10.7) | | | | 7 (4.7) | | | | 2 (1.3) | | | 0 (0.0) |
| Item 14. Heavy objects | | 79 (53.0) | | | 41 (27.5) | | | | 19 (12.8) | | | | 8 (5.4) | | | | 2 (1.3) | | | 0 (0.0) |
| Item 15. Short walk | | 102 (68.5) | | | 29 (19.5) | | | | 15 (10.1) | | | | 3 (2.0) | | | | 0 (0.0) | | | 0 (0.0) |
| Item 16. Long walk | | 80 (53.7) | | | 40 (26.8) | | | | 18 (12.1) | | | | 7 (4.7) | | | | 4 (2.7) | | | 0 (0.0) |
| Item 17. Brisk walk | | 81 (54.4) | | | 37 (24.8) | | | | 18 (12.1) | | | | 9 (6.0) | | | | 4 (2.7) | | | 0 (0.0) |
| Item 18. Walk up | | 70 (47.0) | | | 51 (34.2) | | | | 17 (11.4) | | | | 10 (6.7) | | | | 1 (0.7) | | | 0 (0.0) |
| **Emotion and lifestyle items** | | **Not at all** | | | | **A little** | | | | | **Quite a lot** | | | | | **Very much** | | | | **Missing** |
| Item 19. Worry | | 22 (14.8) | | | | 74 (49.7) | | | | | 40 (26.8) | | | | | 13 (8.7) | | | | 0 (0.0) |
| Item 20. Feel down | | 54 (36.2) | | | | 72 (48.3) | | | | | 17 (11.4) | | | | | 6 (4.0) | | | | 0 (0.0) |
| Item 21. Feel angry | | 85 (57.0) | | | | 49 (32.9) | | | | | 11 (7.4) | | | | | 4 (2.7) | | | | 0 (0.0) |
| Item 22. Feel judged | | 108 (72.5) | | | | 32 (21.5) | | | | | 9 (6.0) | | | | | 0 (0.0) | | | | 0 (0.0) |
| Item 23. Relationships | | 114 (76.5) | | | | 26 (17.4) | | | | | 8 (5.4) | | | | | 1 (0.7) | | | | 0 (0.0) |
| Item 24. Everyday activities | | 107 (71.8) | | | | 30 (20.1) | | | | | 9 (6.0) | | | | | 3 (2.0) | | | | 0 (0.0) |
| Item 25. Family life | | 115 (77.2) | | | | 29 (19.5) | | | | | 3 (2.0) | | | | | 2 (1.3) | | | | 0 (0.0) |
| Item 26. Worry to family | | 69 (46.3) | | | | 63 (42.3) | | | | | 14 (9.4) | | | | | 3 (2.0) | | | | 0 (0.0) |
| Item 27. Intimacy | | 116 (77.9) | | | | 22 (14.8) | | | | | 7 (4.7) | | | | | 4 (2.7) | | | | 0 (0.0) |
| Item 28. Socialise | | 105 (70.5) | | | | 28 (18.8) | | | | | 14 (9.4) | | | | | 2 (1.3) | | | | 0 (0.0) |
| Item 29. Spare time | | 99 (66.4) | | | | 39 (26.2) | | | | | 9 (6.0) | | | | | 2 (1.3) | | | | 0 (0.0) |
| Item 30. Work or study | | 104 (69.8) | | | | 36 (24.2) | | | | | 7 (4.7) | | | | | 2 (1.3) | | | | 0 (0.0) |
| Item 31. Food restriction | | 43 (28.9) | | | | 72 (48.3) | | | | | 28 (18.8) | | | | | 6 (4.0) | | | | 0 (0.0) |

NASH = non-alcoholic steatohepatitis.

Table S4. Exploratory Factor Analysis Factor Loadings for NASH-CHECK Symptoms Items, Activity Limitations Items, and Emotion and Lifestyle Items

| NASH-CHECK Items | Standardized Factor Loadings | | | |
| --- | --- | --- | --- | --- |
| **Symptoms Items^1^ (3-Factor model)** | **Factor 1** | **Factor 2** | | **Factor 3** |
| Pain | **0.955*** | — | | — |
| Bloating | **0.422*** | 0.393* | | — |
| Fatigue | — | **0.954*** | | — |
| Rest | — | **0.819*** | | — |
| Sleep | 0.188 | 0.254 | | 0.239 |
| Focusing | — | — | | **0.679*** |
| Thinking | — | — | | **1.060*** |
| Conversation | — | — | | **0.862*** |
| Forgetful | — | 0.309* | | **0.506*** |
| Itchy skin | **0.392** | — | | — |
| **Activity Limitations Items^2^ (2-Factor Model)** | **Factor 1** | | **Factor 2** | |
| Bending | — | | **0.933*** | |
| Light chores | — | | **0.997*** | |
| Heavy chores | — | | **0.743*** | |
| Heavy objects | — | | **0.904*** | |
| Short walk | **0.947*** | | — | |
| Long walk | **1.031*** | | — | |
| Brisk walk | **0.620*** | | 0.346* | |
| Walk up | **0.738*** | | — | |
| **Emotions and Lifestyle Items^2^ (2-Factor Model)** | **Factor 1** | | **Factor 2** | |
| Worry | **0.721*** | | — | |
| Feel down | **0.904*** | | — | |
| Angry | **0.667*** | | — | |
| Feeling judged | **0.499*** | | — | |
| Relationships | — | | **0.818*** | |
| Everyday activities | — | | **0.870*** | |
| Family life | — | | **0.923*** | |
| Worry to family | 0.368* | | **0.418*** | |
| Intimacy | — | | **0.927*** | |
| Socialise | — | | **0.705*** | |
| Spare time | — | | **0.839*** | |
| Work or study | — | | **0.925*** | |
| Food restriction | — | | **0.305** | |

* *P* < 0.05 for H_0_: Loading = 0.

EFA = exploratory factor analysis; NASH = non-alcoholic steatohepatitis.

Notes: Models presented are the best fitting models from the EFA using Part B baseline data (N = 103).

^1^ Exploratory factor analysis was conducted using oblique quartimin rotation and maximum likelihood estimation with robust standard errors.

^2^ Exploratory factor analysis was conducted using oblique quartimin rotation and weighted least-square estimation with adjusted mean and variance.

— Loadings of magnitude < 0.3 are not presented, except for item 5 (sleep).

**Bold** denotes the highest factor loadings ≥ 0.3 for each item.

Table S5. Construct Validity Correlations Between NASH-CHECK Scores and Clinical Assessments at Baseline

| NASH-CHECK Scale Score | Correlation Coefficient^a^ | | | | | | |
| --- | --- | --- | --- | --- | --- | --- | --- |
|  | NAS^b^ | NAFLD Fibrosis Score | ELF Score | ALT Level | AST Level | GGT Level | Hepatic Fat |
| Abdominal Pain | 0.01 | 0.03 | 0.00 | 0.04 | 0.04 | 0.02 | −0.01 |
| Abdominal Bloating | 0.03 | 0.04 | −0.04 | −0.01 | −0.02 | −0.00 | 0.06 |
| Fatigue | −0.03 | 0.01 | −0.08 | −0.01 | −0.04 | −0.05 | 0.07 |
| Sleep | 0.12 | 0.17 | 0.10 | −0.05 | 0.01 | −0.06 | 0.02 |
| Itchy Skin | −0.02 | −0.06 | −0.06 | −0.01 | −0.00 | −0.06 | −0.01 |
| Cognitive Symptoms | 0.12 | 0.04 | −0.03 | 0.01 | 0.03 | −0.13 | 0.10 |
| Activity Limitations | 0.07 | 0.18 | 0.04 | −0.15 | −0.12 | −0.10 | 0.08 |
| Emotional Impact | 0.07 | 0.03 | −0.07 | −0.03 | −0.06 | 0.02 | 0.02 |
| Social Impact | 0.08 | 0.01 | −0.09 | −0.02 | −0.05 | −0.09 | 0.13 |

ALT = alanine aminotransferase; AST = aspartate aminotransferase; ELF = enhanced liver fibrosis; GGT = gamma-glutamyl transferase; NAFLD = nonalcoholic fatty liver disease; NASH = nonalcoholic steatohepatitis; NAS = NAFLD Activity Score.

^a^ Pearson correlation for NAFLD Fibrosis Score, ELF score, ALT level, AST level, GGT level, and hepatic fat; polyserial correlation for NAS.

^b^ Part C only.

Table S6. Responsiveness Correlations Between Changes in NASH-CHECK Scales and Changes in Supporting Patient-Reported Measures From Baseline to Week 48

| **NASH-CHECK Scale** | **Correlation Coefficient** | | | | | | | | | | | |
| --- | --- | --- | --- | --- | --- | --- | --- | --- | --- | --- | --- | --- |
|  | **VAS for Itch** | **VAS for Sleep Disturbance** | **PGIS** | **PGIC** | **CLDQ Fatigue** | **CLDQ Activity** | **CLDQ Emotional Function** | **CLDQ Abdominal Symptoms** | **CLDQ Systemic Symptoms** | **CLDQ Worry** | **CLDQ Total** | **EQ-VAS** |
| Abdominal Pain | 0.08 | 0.20 | 0.31 | −0.16 | −0.28 | −0.22 | −0.22 | −0.44 | −0.26 | −0.09 | −0.33 | −0.23 |
| Abdominal Bloating | 0.08 | 0.16 | 0.33 | −0.24 | −0.56 | −0.32 | −0.43 | −0.72 | −0.50 | −0.12 | −0.57 | −0.11 |
| Fatigue | 0.11 | 0.22 | 0.48 | −0.26 | −0.68 | −0.39 | −0.48 | −0.53 | −0.46 | −0.26 | −0.60 | −0.07 |
| Sleep | 0.17 | 0.48 | 0.41 | −0.47 | −0.53 | −0.23 | −0.64 | −0.48 | −0.50 | −0.10 | −0.52 | −0.32 |
| Itchy skin | 0.75 | 0.34 | 0.23 | −0.11 | −0.14 | −0.02 | −0.29 | −0.19 | −0.26 | 0.02 | −0.18 | 0.05 |
| Cognitive Symptoms | −0.05 | 0.26 | 0.44 | −0.29 | −0.57 | −0.40 | −0.67 | −0.49 | −0.53 | −0.36 | −0.63 | −0.18 |
| Activity Limitations | −0.08 | 0.12 | 0.48 | −0.31 | −0.59 | −0.42 | −0.53 | −0.43 | −0.48 | −0.30 | −0.58 | −0.30 |
| Emotional Impact | −0.01 | −0.01 | 0.23 | −0.19 | −0.35 | −0.35 | −0.40 | −0.27 | −0.39 | −0.54 | −0.48 | −0.29 |
| Social Impact | 0.02 | 0.05 | 0.30 | −0.24 | −0.36 | −0.36 | −0.41 | −0.28 | −0.30 | −0.52 | −0.47 | −0.34 |

CLDQ = Chronic Liver Disease Questionnaire; EQ-VAS = EQ-5D visual analogue scale; NASH = non-alcoholic steatohepatitis; PGIC = Patient Global Impression of Change; PGIS = Patient Global Impression of Severity; PRO = patient-reported outcome; VAS = visual analogue scale.

Note: Pearson correlations for VAS for itch, VAS for sleep disturbance, CLDQ scales, and EQ-VAS. Polyserial correlations for PGIS and PGIC.

Change in NASH-CHECK scores calculated as week 48 minus baseline; negative change scores indicate improvement.
